# Synaptic secretion from human natural killer cells is diverse and includes supramolecular attack particles

**DOI:** 10.1073/pnas.2010274117

**Published:** 2020-09-08

**Authors:** Ashley R. Ambrose, Khodor S. Hazime, Jonathan D. Worboys, Olatz Niembro-Vivanco, Daniel M. Davis

**Affiliations:** ^a^The Lydia Becker Institute of Immunology and Inflammation, University of Manchester, M13 9NT Manchester, United Kingdom

**Keywords:** natural killer cells, immune synapse, supramolecular attack particles

## Abstract

Natural killer (NK) cells form immune synapses to ascertain the state of health of cells they encounter. If a target cell triggers NK cell cytotoxicity, lytic granules containing proteins including perforin and granzyme B, are secreted into the synaptic cleft inducing target cell death. Secretion of these proteins also occurs from activated cytotoxic T lymphocytes (CTLs) where they have recently been reported to complex with thrombospondin-1 (TSP-1) in specialized structures termed supramolecular attack particles (SMAPs). Here, using an imaging method to define the position of each NK cell after removal, secretions from individual cells were assessed. NK cell synaptic secretion, triggered by ligation of NKp30 or NKG2D, included vesicles and SMAPs which contained TSP-1, perforin, and granzyme B. Individual NK cells secreted SMAPs, CD63+ vesicles, or both. A similar number of SMAPs were secreted per cell for both NK cells and CTLs, but NK cell SMAPs were larger. These data establish an unexpected diversity in NK cell synaptic secretions.

## Significance

Cytotoxic immune cells kill cancer and virally infected cells through secretion of perforin and granzymes at immune synapses. It has recently been shown that cytotoxic T cells secrete membraneless protein structures, termed SMAPs, comprising perforin and granzyme within a shell of the glycoprotein TSP-1. Here, using a novel imaging technique termed "shadow imaging," we quantitatively assessed synaptic secretion from individual human NK cells. Upon ligation of activating receptors, NK cells secreted vesicles as well as membraneless SMAPs containing TSP-1, perforin, and granzyme B. Super-resolution microscopy revealed that NK cell SMAPs were larger than those secreted by T cells. Heterogeneity in NK cell synaptic secretion is likely important for the different effector functions of NK cell subtypes.

Natural killer (NK) cells are vital components of our immune response against virus-infected or cancerous cells. NK cells form an immune synapse (IS) with cells they contact and respond according to a balance of stimulatory and inhibitory signals (1). Secretion of granzymes and perforin across the synapse facilitates NK cell cytolysis (2, 3). Within NK cells, perforin and granzymes are packaged inside lytic granules with serglycin, which neutralizes their cytolytic activity (4, 5). How perforin and granzyme are packaged during secretion is poorly understood. Recently, Balint et al. identified SMAPs released from CTLs (6). SMAPs are composed of membraneless complexes of perforin, granzyme B, and TSP-1. Knockdown of TSP-1 reduced CTL-induced killing, implicating an integral role in cytotoxicity. Other molecules are also secreted at synapses within vesicles (7). Here, we present a method, termed shadow imaging, to observe single-cell secretions. We find that SMAPs are also secreted from human NK cells and that these are larger than those from CTLs. Moreover, we report diversity in synaptic secretions by human NK cells, with CD63+ vesicles also contributing to the milieu.

## Materials and Methods

### Cell Isolation and Culture.

Peripheral blood was acquired from the National Health Service blood service (Ethics license: 05/Q0401/108). Peripheral blood mononuclear cells were purified by density gradient centrifugation (Ficoll-Paque Plus; GE Healthcare) with NK cells and CD8^+^ T cells isolated using negative selection microbeads (Miltenyi Biotec). NK cells were cultured (37 °C/5% CO_2_) with 200 U/mL rhIL-2 (Roche), but used when resting 6 d later. CTLs were used immediately after isolation.

### Preparation of Coated Slides and Bilayers.

Eight chamber glass slides (1.5 Lab-Tek II; Nunc) were coated with 0.01% poly-l-lysine (PLL) and dried at 60 °C for 1 h. Slides were coated with His-ICAM-1 (2.5 µg/mL; produced in house) alone or with His-MICA (2.5 µg/mL; Sino Biological), B7-H6-Fc (2.5 µg/mL; R&D Systems), αNKG2A (5 µg/mL; R&D Systems), or αNKp30 (10 µg/mL; P30-15; Biolegend or 210845; R&D Systems) in phosphate-buffered saline (PBS) overnight (4 °C). Bilayers were prepared as previously described (8) and functionalized with His-ICAM-1 (2.5 µg/mL) alone or with His-MICA (2.5 µg/mL), biotinylated-αNKp30 (10 µg/mL; P30-15), or biotinylated-αCD3 (5 µg/mL; OKT3; a gift from Andy Shepherd, GSK).

### IFNγ Enzyme-Linked Immunosorbent Assay.

NK cells (1 × 10^5^) were incubated on coated slides for 16 h (37 °C/5% CO_2_), supernatants aspirated, and cells pelleted at 1,000 *g* for 10 min (4 °C). IFNγ concentration was measured by enzyme-linked immunosorbent assay (DuoSet, R&D Systems) according to manufacturer’s instructions.

### Imaging NK Cell Secretions.

NK cells (1 × 10^5^) were incubated on coated slides for 1 h (37 °C/5% CO_2_), then detached with nonenzymatic cell-dissociation solution (Sigma-Aldrich) for 20 min (37 °C) and washed with PBS. Where indicated, to inhibit exosome secretion, cells were preincubated with 100 nM cambinol (Sigma-Aldrich) for 1 h (37 °C). Slides were blocked with 1% bovine serum albumin (Sigma-Aldrich) and 1% human serum (ThermoFisher Scientific) in PBS for 1 h at room temperature (RT) and stained for 1 h (RT) with mAbs: αTSP-1-AF647 (10 µg/mL; A6.1), αSerglycin-AF647 (10 µg/mL; C-11), αGalectin-1-AF647 (10 µg/mL; C-8) (all Santa Cruz Biotechnology), αCD63-AF647 (10 µg/mL; H5C6; BioLegend), αGranzyme B-AF647 (10 µg/mL; GB11; BioLegend), or αPerforin-AF488 (2.5 µg/mL; dG9; Biolegend). For coordinate-based colocalization positive controls, slides were first stained with αPerforin-AF488 (2.5 µg/mL; dG9; Biolegend) for 1 h at RT, washed with PBS, and then stained with a goat anti-mouse IgG2b secondary antibody conjugated to AF647 (ThermoFisher Scientific) for 1 h at RT. Wheat germ agglutinin (WGA) conjugated to CF568 (2 µg/mL; Biotium) or AF647 (2 µg/mL; ThermoFisher Scientific) was used to stain glycoproteins and DiD (1 µM; ThermoFisher Scientific) to mark membrane phospholipids. Samples were washed with PBS and imaged with 488/561/647 nm lasers on an Eclipse Ti inverted microscope (Nikon) using an Apo total internal reflection (TIRF) 100× 1.49 numerical aperture (NA) oil objective, or using 488/642 nm lasers on a SR GSD (ground state depletion) microscope (Leica Biosystems) using a 160× 1.43 NA oil objective. Images were analyzed within ImageJ (9).

### Shadow Imaging.

NK cells were prepared as before, but prior to cell detachment, slides were washed with PBS, stained with αICAM-1-AF488 (HCD54; 5 µg/mL; BioLegend) for 1 min, and washed with PBS. Cells were detached and blocked as before then stained with αPerforin-AF647 (2.5 µg/mL; dG9; Biolegend) for 1 h and washed with PBS (all at RT). Cells were imaged by TIRF (Leica SR GSD microscope) using a 160× 1.43 NA oil objective.

### Stochastic Optical Reconstruction Microscopy.

Stochastic optical reconstruction microscopy (STORM) was performed in TIRF mode on a Leica SR GSD microscope, on slides stained as above, using 488 nm or 642 nm lasers for 7,000 frames (11 ms/frame). STORM datasets were analyzed using ThunderSTORM (10) as previously described (11).

### Statistical Analysis.

Statistical analysis was performed using Prism (GraphPad Software; v8.4.2) with specific analyses detailed in the figure legends. All data presented as mean ± SD unless stated.

## Results

### Imaging Reveals Heterogeneous Secretions across the NK Cell Immune Synapse.

**Fig. 1.**
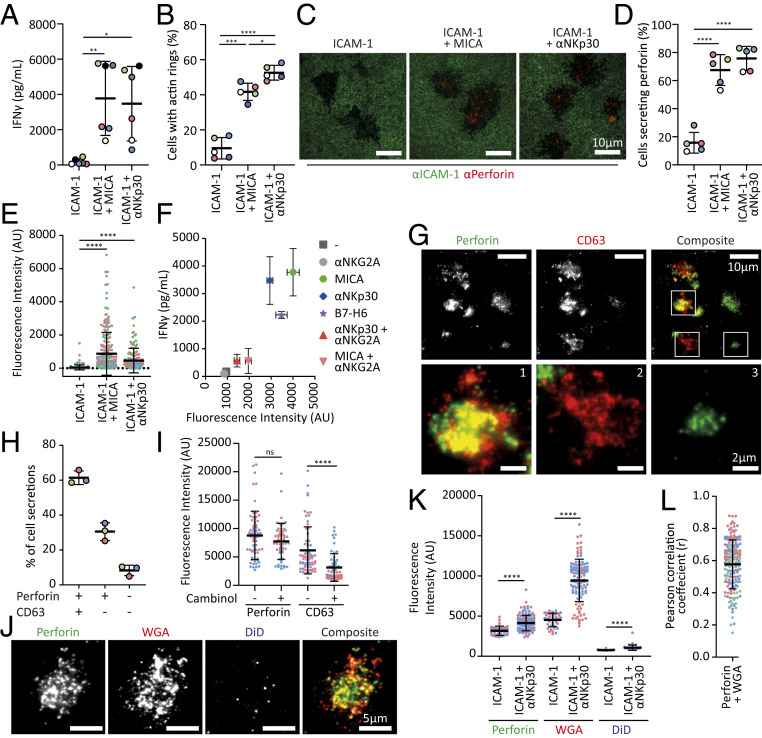
NK secretions at the immune synapse are heterogeneous. (*A*–*E*) NK cells incubated on slides coated with ICAM-1, ICAM-1 + MICA, or ICAM-1 + αNKp30. (*A*) IFNγ secreted from NK cells [*n* = 6; Kruskal–Wallis test]. (*B*) F-actin ring formation on coated slides [*n* = 5; one-way ANOVA]. (*C*) TIRF imaging of perforin and NK cell shadows following cell detachment. (*D*) Percentage of cells secreting perforin [*n* = 5; one-way ANOVA]. (*E*) Intensity of secreted perforin/cell [*n* = 3; Kruskal–Wallis test]. (*F*) Intensity of secreted perforin against IFNγ secretion from NK cells incubated on ICAM-1 plus indicated ligands [*n* = 2–5; mean ± SEM]. (*G*–*L*) NK cells activated with αNKp30. (*G*) TIRF imaging of perforin and CD63 secretions following NK cell detachment. *Bottom* shows expanded views of boxed regions in the composite image. (*H*) Percentage of cells secreting perforin, CD63, or both. (*I*) Intensity of secreted perforin and CD63 with and without 100 nM cambinol [*n* = 2; ≥20 cells/donor; Mann–Whitney *U* test]. (*J*) TIRF images of secretions stained for perforin, WGA, and DiD as indicated. (*K*) Intensity of secreted perforin, WGA, and DiD (*n* = 2; ≥20 cells/donor; Mann–Whitney *U* test). (*L*) Pearson’s correlation coefficient of WGA compared to perforin in secretions (*n* = 3). Cells from individual donors are color coded. Mean ± SD unless stated. * = *P* ≤ 0.05, ** = *P* ≤ 0.01, *** = *P* ≤ 0.001, and **** = *P* ≤ 0.0001.

Initially, we imaged the synaptic secretions of NK cells following stimulation of surface-expressed activating receptors. NK cells were incubated on slides coated with PLL followed by either ICAM-1, ICAM-1 + MICA (a ligand for the activating receptor NKG2D), or ICAM-1 + αNKp30 (a stimulatory mAb). NK cell activation on stimulatory surfaces was confirmed by significant IFNγ release (Fig. 1*A*) and the formation of stable synapses, indicated by assembly of a dense ring of F-actin at cell-slide contact (Fig. 1*B*).

We have previously imaged perforin secretion from human NK cells (12). Here, to investigate the diversity of individual NK cell responses, we developed a method which we term shadow imaging. By introducing a pulsed stain for ICAM-1, NK cells could be detached while leaving a shadow where each had interacted with the slide (Fig. 1*C*). Paired with staining for perforin, this demonstrated that a small fraction (15.8 ± 7.4%) of NK cells secreted detectable amounts of perforin on slides coated with ICAM-1 only. Activation significantly increased the proportion of cells secreting perforin (MICA, 67.5 ± 10.9%; αNKP30, 75.8 ± 8.6%) and the amount secreted per cell (MICA, 21.5-fold and αNKP30, 11.3-fold vs. ICAM-1 alone) (Fig. 1 *D* and *E*). Secretion of IFNγ and perforin from NK cells incubated on slides coated with B7-H6, the cognate ligand for NKp30, was similar to that triggered by αNKp30 mAb (Fig. 1*F*). Furthermore, when both activating (MICA or αNKp30) and inhibitory (αNKG2A) receptors were simultaneously engaged, IFNγ and perforin secretions were reduced (Fig. 1*F*).

Alongside cytotoxic molecules, vesicles can be secreted across the IS as observed for helper T cells (13). Staining secretions from αNKp30-activated NK cells for the exosomal marker CD63 alongside perforin (Fig. 1*G*), revealed that 61.4 ± 3.8% of cells secreted both perforin and CD63, 30.5 ± 5.1% secreted only perforin, and 8.3 ± 3.0% secreted only CD63 (Fig. 1*H*). The exosomal inhibitor cambinol significantly reduced the amount of CD63 detected without affecting the amount of perforin released (Fig. 1*I*). Thus, perforin release occurs independently of exosome secretion. Secreted perforin did not associate with DiD, a lipophilic membrane stain (Fig. 1 *J* and *K*) but strongly colocalized with WGA, marking glycoproteins (Fig. 1 *J* and *L*). This is consistent with secreted perforin being organized in membraneless SMAPs (6). Thus, there is unexpected diversity in synaptic secretion by human NK cells, which includes protein complexes and vesicles.

### TSP-1 and Perforin Colocalize in NK Secretions Resembling CTL Secreted SMAPs.

**Fig. 2.**
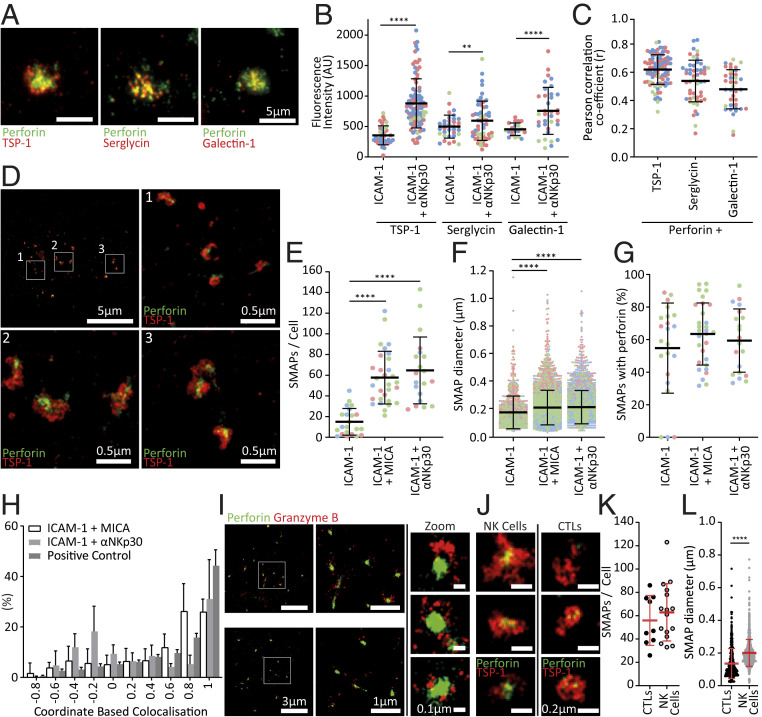
NK cells secrete SMAPs upon activation. (*A*) TIRF microscopy of secretions from αNKp30-activated and detached NK cells. (*B*) Intensity of secreted TSP-1, serglycin, and galectin-1. (*C*) Pearson’s correlation coefficient for TSP-1, serglycin, and galectin-1 compared to perforin (*B* and *C*; *n* = 3; one-way ANOVA). (*D*–*H*) STORM imaging and quantification of perforin and TSP-1 secretions from MICA-activated NK cells. (*D*) Representative STORM images. (*E*) Number of SMAPs secreted/cell. (*F*) Diameter of SMAPs. (*G*) Percentage of SMAPs that contain a detectable level of perforin. (*H*) Coordinate-based colocalization comparing perforin to TSP-1. (*E*–*H*; *n* = 3; ≥5 cells/donor; Kruskal–Wallis test). (*I*) STORM imaging of perforin and granzyme B in secretions from MICA-activated and detached NK cells. (*J*) STORM images of SMAPs secreted from MICA-activated NK cells and αCD3-activated CTLs. (*K*) Number of SMAPs secreted/cell. (*L*) Diameter of SMAPs. Cells from individual donors are color coded. Mean ± SD ** = *P* ≤ 0.01 and **** = *P* ≤ 0.0001.

Balint et al. recently demonstrated that perforin is secreted from CTLs in complex with TSP-1 and granzyme B (6). Prior work also established that serglycin and galectin-1 are important components of lytic granules from which perforin is released (4, 14). Here, NK cell secretions of perforin were observed alongside TSP-1, serglycin, and galectin-1 (Fig. 2*A*). Some secretion of these proteins occurred when NK cells contacted a slide containing only ICAM-1, but this was increased by bona fide NK cell activation via NKp30 (Fig. 2*B*). Perforin colocalized with all three other proteins, especially TSP-1 (*r* = 0.62 ± 0.11; Fig. 2*C*).

The nanoscale distribution of TSP-1 and perforin was assessed using the super-resolution microscopy method STORM (Fig. 2*D*). This established that TSP-1 complexes had a diameter of ∼210 nm and usually contained perforin (Fig. 2 *E*–*G*). Coordinate-based colocalization showed TSP-1 and perforin were strongly associated with 30% of localized molecules having a colocalization of ≥0.8 for activated NK cells (Fig. 2*H*). Granzyme B was also directly associated with perforin (Fig. 2*I*).

We next compared secretions from NK cells and CTLs on activating planar lipid bilayers (PLBs) (Fig. 2*J*) which offer a more physiological surface than PLL. Each individual cell secreted a similar number of SMAPs (NK cells: 63 ± 25 [MICA] per cell; CTLs: 55 ± 21 per cell [αCD3]; Fig. 2*K*). Intriguingly, we found that NK cell-derived SMAPs were larger (NK cells: 201 ± 81 nm [MICA] and CTLs: 135 ± 91 nm [αCD3]; Fig. 2*L*). Altogether, this establishes significant diversity in synaptic secretions between individual cells as well as different types of cells.

## Discussion

Shadow imaging enabled single-cell assessment of NK cell secretions and is a method which is generalizable to other stimulations and cell types. We observed heterogeneous secretions from NK cells with individual cells secreting vesicles, cytotoxic molecules, or both. The use of PLL-coated surfaces rather than planar lipid bilayers permitted shadow imaging and, by its nonspecific electrostatic interactions, may capture a broader range of synaptic secretions. Recent work identified SMAPs in CTL secretions, which we observe here in NK cells. In both NK cells and CTLs, SMAPs contain a cytolytic protein core, currently known to comprise perforin and granzyme B, surrounded by a glycoprotein shell of TSP-1. In some SMAPs, the TSP-1 shell appeared disrupted, but this may reflect the use of chelating agents in the NK cell detachment solution. Soluble perforin and granzymes are sufficient to induce cell death (15), but complexing within SMAPs may offer a mechanism to increase localized perforin concentrations in target cell membranes, or to prevent toxic proteins leaking out of the synaptic cleft (16). The discovery of SMAPs establishes a new paradigm for how cytotoxic cells elicit target cell killing, and elucidating the mechanism by which TSP-1 contributes to cytolytic function may have therapeutic potential. Finally, diversity of synaptic secretions by NK cells is likely important in how subpopulations contribute to an immune response.

## Data Availability

All data are included within the paper.
